# Supplementary material for: Gradual Response of Cyanobacterial Thylakoids to Acute High-Light Stress—Importance of Carotenoid Accumulation
Source: Cells. 2021 Jul 28;10(8):1916. doi: 10.3390/cells10081916 (PMC8393233; doi:10.3390/cells10081916)
Supplement: Supplementary file 1 [file cells-10-01916-s001.zip › cells-1271409-supplementary.pdf]

SUPPLEMENTAL DATA:

Gradual response of cyanobacterial thylakoids to acute high–light stress – importance of carotenoids accumulation

Myriam Canonico, Grzegorz Konert, Aurélie Crepin, Barbora Šedivá and Radek Kaňa

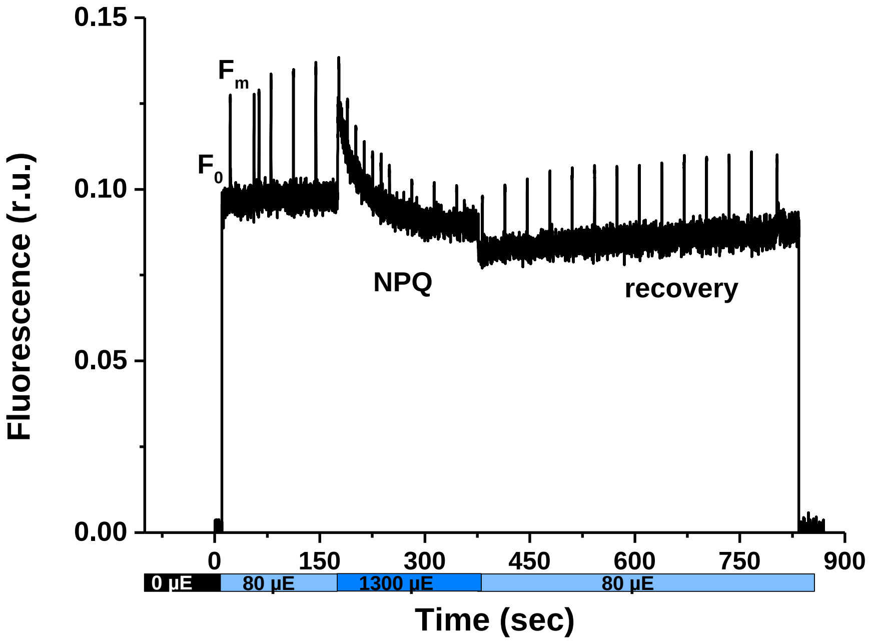

Figure S1. Example of a measurement using Dual Pam.

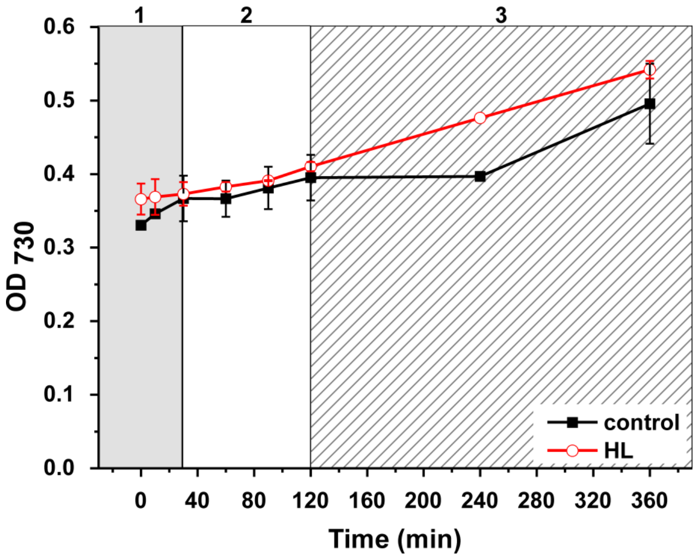

Figure S2. Optical density of *Synechocystis PSI–YFP* culture measured at 730 nm. Black line = control; red line = HL. Data and SD represent average of 4 independent replicates. Background and associated numbers represent phases we detected in the treatment: grey: phase 1 (fast response); white: phase 2 (intermediate); striped: phase 3 (slow acclimation).

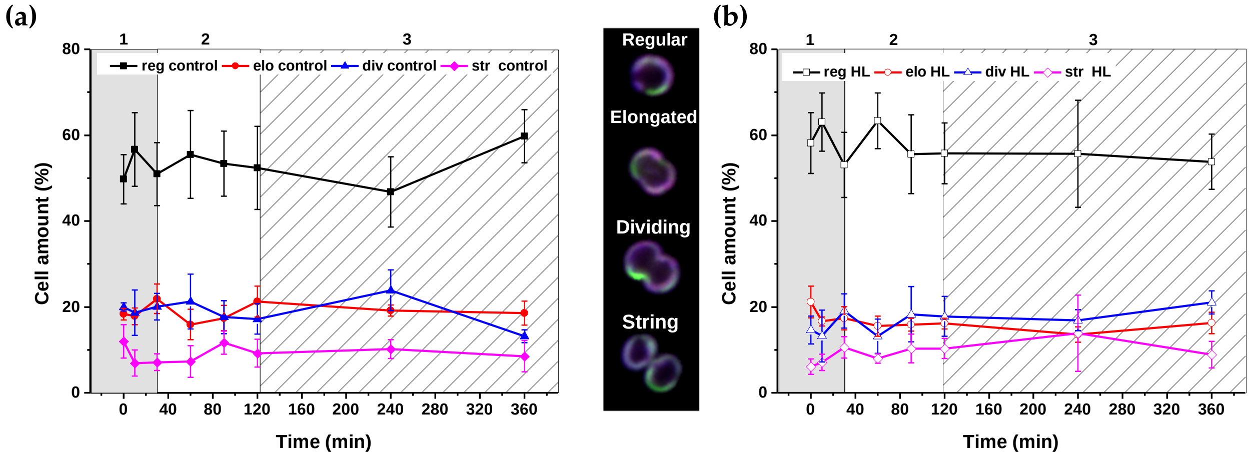

Figure S3. Cell type counts of *Synechocystis PSI−YFP*. Relative occurrence of 4 types of cell shapes: reg = regular; elo = elongated; div = dividing; str = string. Control **(a)** and HL **(b)** conditions. Data and SD represent averages of 6 biological experiments. Background and associated numbers represent phases we detected in the treatment: grey: phase 1 (fast response); white: phase 2 (intermediate); striped: phase 3 (slow acclimation).

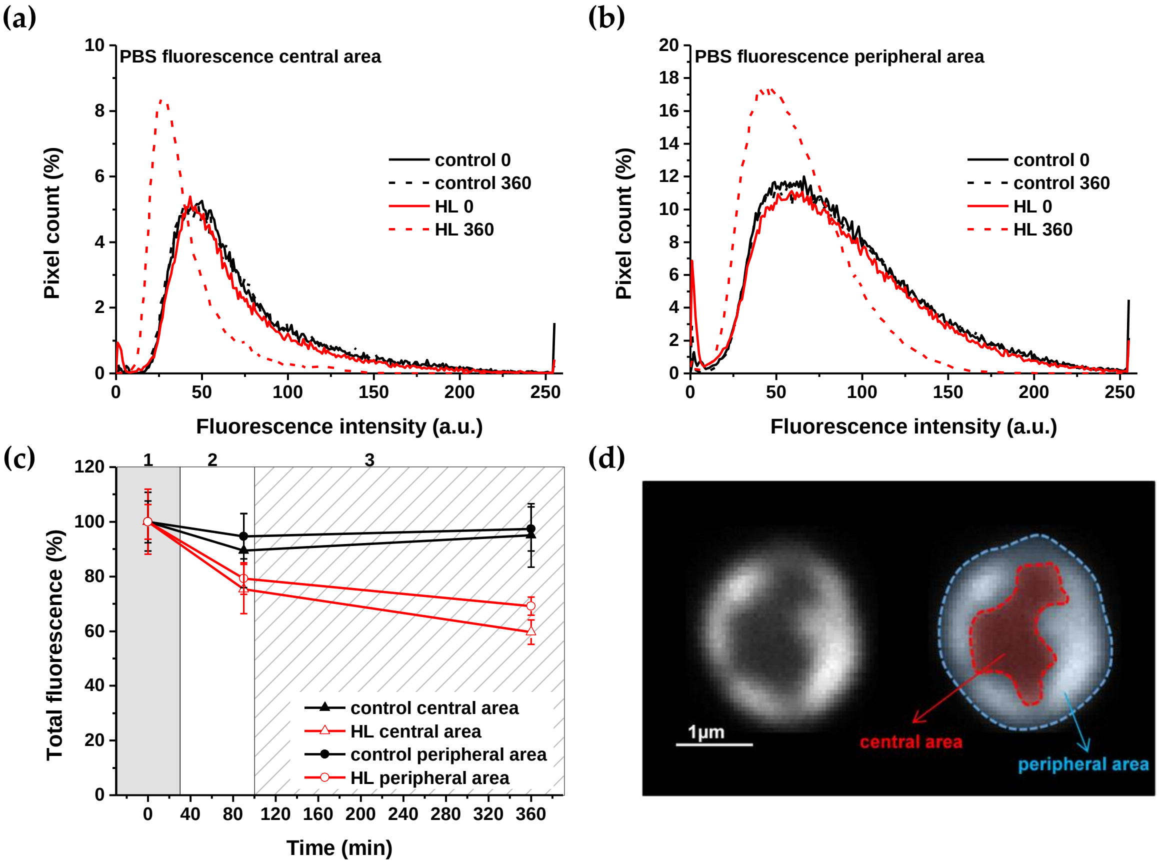

Figure S4. Phycobilisomes fluorescence emission measured in the thylakoid membrane (TM) area and in the central cell area outside of the TM. Histogram of fluorescence intensities in cells were calculated in two regions, thylakoid membrane area **a)** and central cell area **b)** for n= 5844 cells. Individual cells values were normalized to total emission detected in inner or outer region, followed by averaging cells from corresponding conditions. PBS fluorescence of *Synechocystis PSI–YFP*. **c)** Total fluorescence (percentage) of control (black) and HL (red) cells. (**d**) Representative image showing central and peripheral areas in the cell.

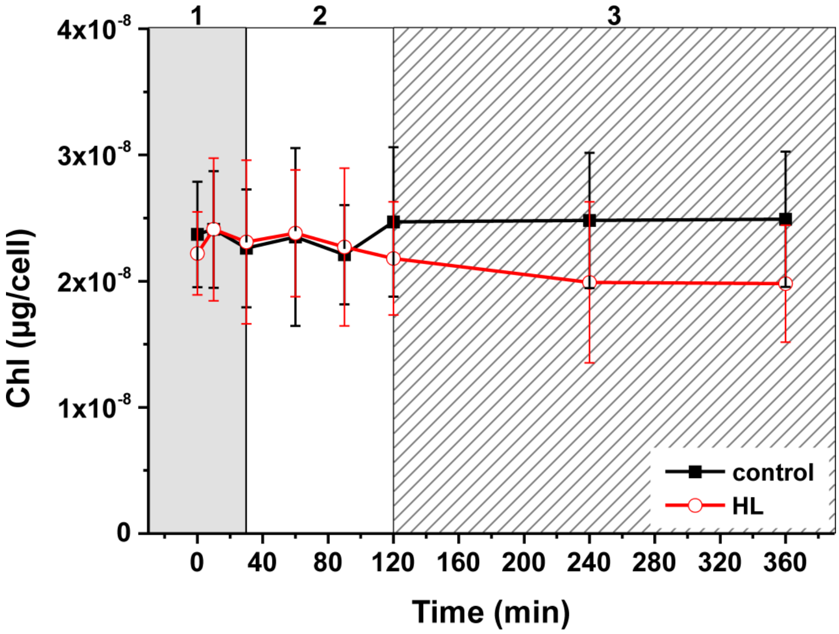

Figure S5. Chlorophyll concentration per cell. Chlorophyll concentration was calculated according to *Ritchie,* 2006 [1] and then normalized by total cell number per time point. Black line= control; red line = HL. Data and SD represent average of 4 independent biological repetitions.

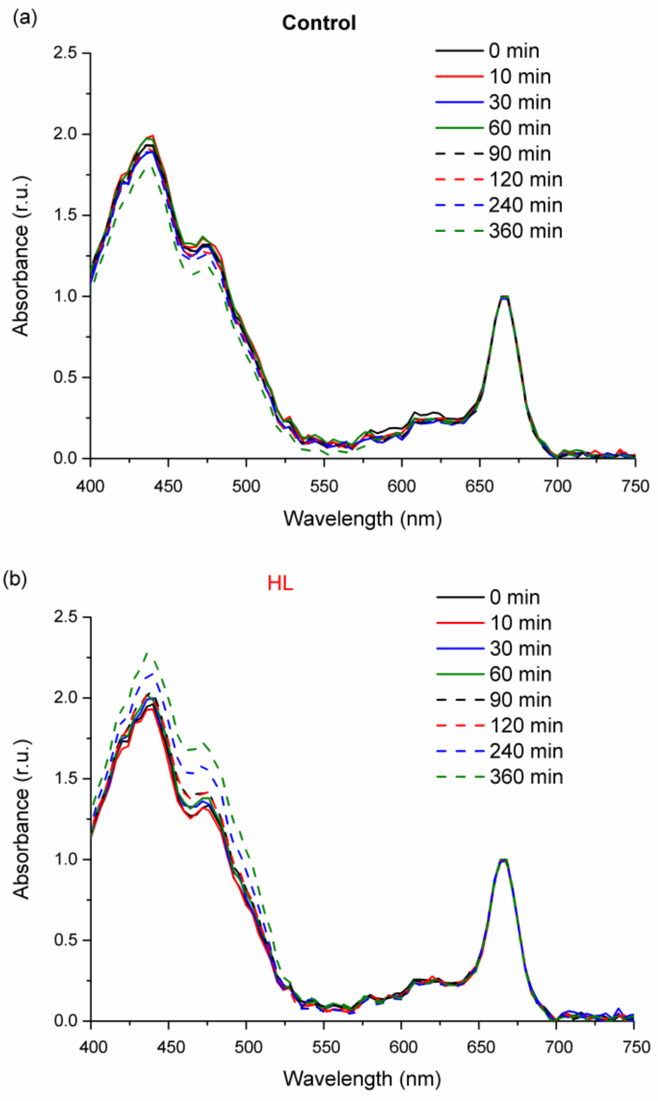

Figure S6. Absorption spectra of extracted pigments. Straight black line = time 0 minutes; straight red line = time 10 minutes; straight blue line = time 30 minutes; straight green line = time 60 minutes; dashed black line = time 90 minutes; dashed red line = time 120 minutes; dashed blue line time 240 minutes; dashed green light = time 360 minutes. **(a)** control **(b)** HL. Data were normalized to Chl peak at 665 nm.

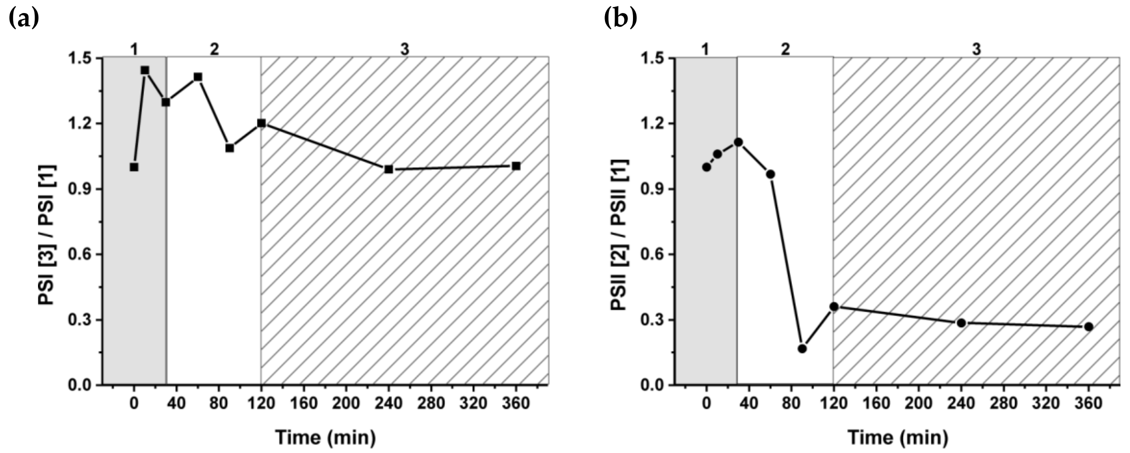

Figure S7. Protein composition analysis from CN gel of HL treated culture. **(a)** PSI [3] / PSI [1] **(b)** PSII [2] / PSII [1].

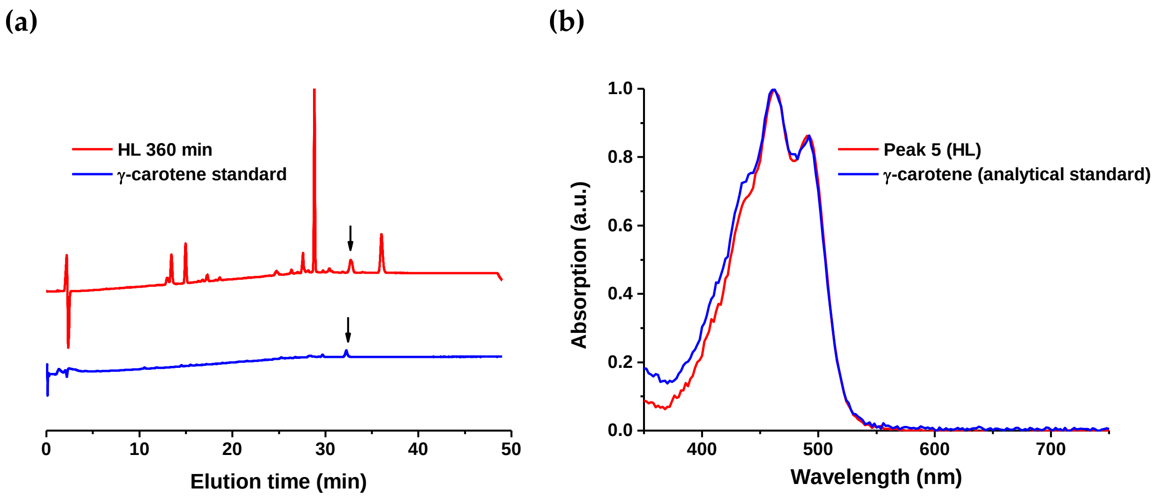

Figure S8. Identification of ɣ-carotene. **(a)** Elution time of pigments of HL sample at 360 min (red line) and ɣ-carotene analytical standard (blue line). Arrows indicate ɣ-carotene. **(b)** Absorption spectra comparison of peak n°5 (red line – see main text) and ɣ-carotene analytical standard (blue line).

1. Ritchie, R.J., *Consistent sets of spectrophotometric chlorophyll equations for acetone, methanol and ethanol solvents.* Photosynth Res, 2006. **89**(1): p. 27-41.
